# Immunoglobulin A Isotype of Antiphospholipid Antibodies Does Not Provide Added Value for the Diagnosis of Antiphospholipid Syndrome in a Chinese Population

**DOI:** 10.3389/fimmu.2020.568503

**Published:** 2020-10-05

**Authors:** Chaojun Hu, Xi Li, Jiuliang Zhao, Qian Wang, Mengtao Li, Xinping Tian, Xiaofeng Zeng

**Affiliations:** ^1^Department of Rheumatology, Peking Union Medical College Hospital, Peking Union Medical College & Chinese Academy of Medical Sciences, Key Laboratory of Rheumatology & Clinical Immunology, Ministry of Education, Beijing, China; ^2^National Clinical Research Center for Dermatologic and Immunologic Diseases (NCRC-DID), Beijing, China; ^3^Department of Clinical Laboratory, First Affiliated Hospital of Guangxi Medical University, Nanning, China

**Keywords:** IgA isotype of antiphospholipid antibodies, IgA isotype of anticardiolipin antibodies, antiphospholipid syndrome, diagnostic value, IgA isotype of anti-β2 glycoprotein-I

## Abstract

**Objective:**

Antiphospholipid syndrome (APS) is characterized by the presence of anti-phospholipid (aPL) antibodies. However, the relationship between the immunoglobulin (Ig) A isotype of aPL positivity and its clinical utility in APS diagnosis is controversial. Presently, we determine the clinical utility of IgA–aPL from consecutive patients in a large cohort from the Chinese population and patients with APS whose aPL profiles were obtained.

**Methods:**

The detection of anticardiolipin (aCL) and anti-β_2_ glycoprotein-Ⅰ (aβ_2_GPⅠ) antibodies of the IgA/IgG/IgM isotype by paramagnetic particle chemiluminescent immunoassay was carried out in sera from 7293 subjects. 153 primary APS (PAPS) patients and 59 patients with secondary APS (SAPS) were included in this study.

**Results:**

In total, 1,082 out of 7,293 (2.55%) subjects had a positive IgA–aPL test, and the prevalence of isolated IgA–aPL was 0.29% (21/7,293) in the general population. The prevalence of IgA–aPL in the PAPS patients was 12.42% (19/153); however, only one patient (0.65%) presented with isolated IgA–aPL. Fifty (25.9%) of the SAPS had IgA–aPL, none of whom lacked IgG/IgM–aPL. The combination of the IgA isotype and the IgG/IgM isotype did not increase the diagnostic performance when compared with the IgG/IgM isotype of aCL or aβ_2_GPⅠ, respectively. IgA–aPL was not associated with clinical manifestation in patients with APS.

**Conclusion:**

Isolated IgA–aPL is rare in the general population as well as in patients with APS. Whether in the laboratory or in clinical practice, the presence of IgA–aPL does not provide added value for the diagnosis of APS in the Chinese population.

## Introduction

Antiphospholipid syndrome (APS) is an autoimmune disease with characteristic features of recurrent thrombosis and/or obstetric complications accompanied with the persistent presence of antiphospholipid antibodies (aPL) (1). aPL are initially produced by B lymphocytes and are connected to β_2_ glycoprotein-I (β_2_GPI) in endothelial cells, which subsequently causes a series of harmful immune responses through a variety of mechanisms, including activation of inflammatory cells and endothelial cells leading to inflammation and thrombosis as well as interference with trophoblasts and decidual cells leading to pregnancy-related morbidity (2–6). According to the 2006 update of the International consensus statement on classification criteria for APS, the presence of lupus anticoagulant (LA) and/or the immunoglobulin (Ig) G and/or IgM isotypes of anticardiolipin antibodies (aCL) and/or anti-β_2_ glycoprotein-I (aβ_2_GPI) indicate APS (1).

The patients with a clinical manifestation that was highly suggestive of APS but persistently negative for routine aPL (IgG/IgM–aCL, IgG/IgM–aβ_2_GPI, and LA) were defined as having seronegative APS (7). Currently, researchers are trying to discover other nonstandard antibodies to improve the diagnosis of the so-called seronegative APS, such as the IgA isotype of aPL, anti-β_2_GPI domain I, antiphosphatidylethanolamine, and antiphosphatidylserine/prothrombin antibodies (8–10). Among them, IgA–aPL has shown much promise. On the one hand, it has been claimed that detection of IgA–aPL is significantly correlated with thrombosis and obstetric complications (10), and the presence of IgA–aβ_2_GPI in people with no history of thrombosis events is an independent risk factor for the development of these types of events (11–14). On the other hand, IgA–aPL (IgA–aCL and IgA–aβ_2_GPI) have been classified as one of the laboratory classification criteria for systemic lupus erythemathosus (SLE) (15) and can be used to distinguish seronegative APS from SLE (16, 17). Based on these findings, IgA–aPL seems to be useful for the diagnosis of APS.

However, whether IgA–aPL has the potential to become one of the diagnostic criteria is controversial. For instance, researchers have shown no association between IgA–aPL and clinical manifestations of APS (18, 19). In addition, the detection of IgA–aPL does not increase the diagnostic sensitivity of APS (20) or help diagnose patients with APS-associated SLE (21). Furthermore, the incidence rate of patients with isolated IgA–aPL who experience APS events has been reported to be as low as 3.1% per year (13). Indeed, IgA–aPL is usually accompanied with the IgG/IgM isotype, but the follow-up evidence in patients with isolated IgA–aPL is insufficient. It should be emphasized that the 14th International Congress on Antiphospholipid Antibodies Task Force pointed out that the evidence in available data from IgA aPL was level III-low quality evidence (22). Because of this controversy, researchers have not reached a consensus regarding whether or not they recommend IgA–aPL as one of the criteria for diagnosing APS; therefore, large-scale and high-quality research should be further developed to reveal the relationship between IgA–aPL and the diagnosis of APS.

The aims of this study were to evaluate the prevalence of aCL and aβ_2_GPI according to isotype in a large cohort of patients consecutively referred to the Rheumatology Laboratory as well as the clinical and diagnostic value of the IgA isotype of aPL in the exploration of APS. The associations between IgA–aPL and APS-related clinical manifestations were also determined.

## Materials and Methods

### Subjects and Patients

This retrospective cross-sectional study included 7,293 consecutive subjects who had their aPL profile determined at the Key Laboratory between May 2019 and December 2019. Duplicate patients were removed and for patients with multiple aPL profiles determined during this period, only the first test results were used in this study. This laboratory belongs to the Department of Rheumatology, Peking Union Medical College Hospital (PUMCH) and the National Clinical Research Center for Dermatologic and Immunologic Diseases (NCRC-DID). NCRC-DID is Ministry of Science and Technology of China authorized research center that have collected data on kinds of rheumatic diseases, including SLE, APS, rheumatoid arthritis (RA), ankylosing spondylitis (AS), and so on. The predecessor is Chinese Rheumatism Data Center (CRDC) (23), NCRC-DID provides real-life data to improve clinical decision-making. All the patients with APS diagnosed according to the 2006 Sydney revised classification criteria (1) who were refereed to our center were enrolled in this period. In addition, 120 healthy volunteers were recruited as healthy controls for aPL profile detection. For each subject, 4 mL of blood was collected with the help of a BD vacutainer without anticoagulants. For the next hour, the blood was allowed to clot and was later centrifuged at 4°C for 5 min at 3,000 rpm. The aPL profile was immediately determined in the separated serum. This study was approved by the Medical Ethics Committee of PUMCH, and all methods were carried out in accordance with the principles stated in the Declaration of Helsinki.

### Detection of the aPL Profile

The levels of IgA/IgG/IgM–aCL and IgA/IgG/IgM–aβ_2_GPI were quantified by a paramagnetic particle chemiluminescent immunoassay using an iFlash 3000 Chemiluminescence Immunoassay Analyzer (YHLO Biotech Co. Ltd., Shenzhen, China). Cut-off values were determined strictly in accordance with the manufacturers. The defined cut-off levels for each isotype were as follows: IgA–aCL, 10 APL-U/mL; IgG–aCL, 10 GPL-U/mL; IgM–aCL, 10 MPL-U/mL; IgA–aβ_2_GPI, 20 AU/mL; IgG–aβ_2_GPI, 20 AU/mL; and IgM–aβ2GPI, 20 AU/mL. The manufacturer’s recommendations were followed carefully.

### Statistical Analysis

The results of normally distributed data are expressed as the mean ± standard deviation. Descriptive data are presented as frequencies for categorical variables. Comparisons between two groups were performed by the Student’s t-test. Receiver operating characteristic (ROC) curves were used to calculate the area under the curve (AUC). Logistic regression analysis was employed to determine associations between aPL isotype positivity and clinical manifestation in patients with APS. SPSS 24.0 (SPSS Statistics for Windows, version 24.0; SPSS Inc., Chicago, IL, USA) was used to perform the statistical analysis of the data in this study, and two-tailed P-values of <0.05 represented statistical significance.

## Results

### Patient Characteristics

Consecutive serum samples from 7,293 subjects were screened for the presence of aCL and aβ_2_GPI antibodies with the IgA, IgG, and IgM isotypes. All patients were Chinese. The mean age was 39.5 ± 16.2 years, and 77.65% were female. The detailed demographic data of the study population are listed in **Table 1**. Most patients screened for aPL were from the Department of Rheumatology (52.45%). Of the 7,293 consecutive subjects, 212 patients with a confirmed diagnosis of APS according to the 2006 Sydney revised criteria of APS (1) registered in the NCRC-DID database system were identified in this study (**Table 2**), including 153 patients with PAPS and 59 patients with SAPS. The prevalence of thrombosis events (χ^2^ = 5.552, *P* = 0.02) was significantly higher in patients with SAPS compared to patients with PAPS. The prevalence of a smoking history (χ^2^ = 5.983, *P* = 0.018) was significantly higher in patients with PAPS compared to patients with SAPS.

**Table 1:** Demographic data of 7,293 consecutive subjects.

	Subjects (n = 7,293)	
	n	%
**Age (years)**		
<15	402	5.51
15–30	1,839	25.22
31–60	4,147	56.86
>60	905	12.41
Age (mean ± SD)	39.5 ± 16.2	
**Sex**		
Male	1,630	22.35
Female	5,663	77.65
**Distribution of patients**		
Dept. of Rheumatology	3,825	52.45
Dept. of Obstetrics and Gynecology	442	6.06
Dept. of Hematology	962	13.19
Dept. of Respiration	60	0.82
Dept. of Gastroenterology	76	1.04
Dept. of Pediatrics	297	4.07
Dept. of Nephrology	323	4.43
Dept. of Cardiology	21	0.29
Dept. of Neurology	236	3.24
Other Departments	1,051	14.41

**Table 2:** Demographic and clinical characteristics of the 153 PAPS and 59 SAPS patients.

	PAPS (n = 153)		SAPS (n = 59)		χ^2^/t	P
	n	%	n	%		
Sex (female)	107	69.93	53	91.38	9.105	
Age (mean ± SD)	36.2 ± 11.3		33.14 ± 9.0		1.867	0.063
Thrombosis event	99	64.71	48	81.36	5.552	0.020
Obstetric complications	58	37.91	19	32.20	0.599	0.524
Smoke	24	15.69	2	3.39	5.983	0.018
Hypertension	14	9.15	9	15.25	1.640	0.221
Coronary artery disease	5	3.268	1	1.69	0.383	0.680
Malignancy	2	1.31	0	0.00	0.779	0.596
Lipids disorders	10	6.54	7	11.86	1.639	0.258
Diabetes mellitus	3	1.96	3	5.08	1.511	0.351

### Prevalence of the IgA/IgG/IgM Isotypes of aCL and aβ_2_GPⅠ Antibodies in a Large Cohort

Based on the aPL isotype-specific antibody results, the overall prevalence of aCL was 10.97%, and the most frequent isotype was IgG–aCL (8.64%). The overall prevalence of aβ_2_GPI was 12.16%, and the most frequent isotype was IgM–aβ2GPI (7.72%). The overall prevalence of IgA–aCL and IgA–aβ_2_GPI were 2.48 and 2.13%, respectively. When the prevalence of each isolated isotype of aPL was analyzed, we found that the positive rates of IgA–aCL and IgA–aβ_2_GPI were very low, accounting for only 0.37 and 0.45%, respectively (**Table 3**).

**Table 3:** Profile features of antiphospholipid antibodies in a large-scale cohort.

	Consecutive subjects (n = 7,293)			
	Total positivity		Isolated positivity	
	n	%	n	%
aCL	800	10.97		
IgA–aCL	181	2.48	27	0.37
IgG–aCL	630	8.64	452	6.20
IgM–aCL	185	2.54	139	1.91
aβ2GPⅠ	886	12.15		
IgA–aβ_2_GPⅠ	155	2.13	33	0.45
IgG–aβ_2_GPⅠ	415	5.69	214	2.93
IgM–aβ2GPⅠ	563	7.72	426	5.84

**Figure 1:**
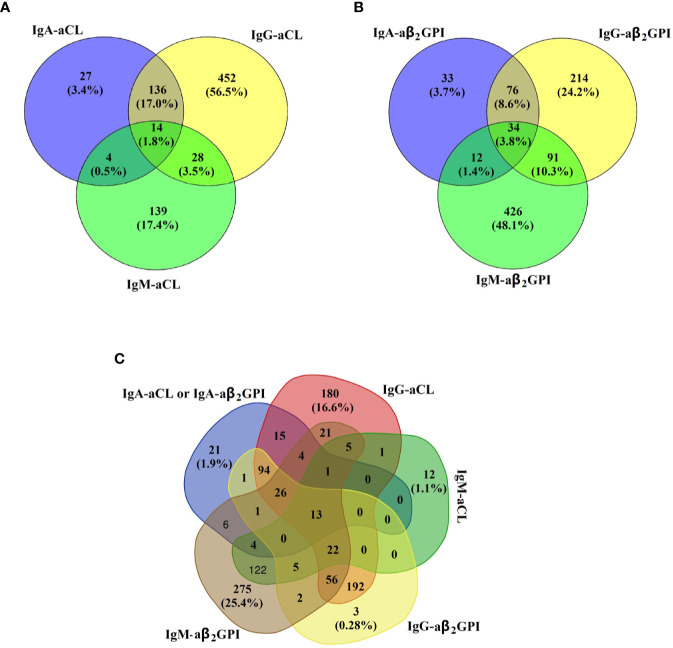
Cross-positivity for **(A)** IgA–aCL, IgG–aCL, and IgM–aCL in 800 aCL-positive subjects; **(B)** IgA–aβ_2_GPⅠ, IgG–aβ_2_GPⅠ, and IgM–aβ2GPI in 886 aβ_2_GPⅠ-positive subjects; and **(C)** IgA–aPL (either IgA–aCL or IgA–aβ_2_GPⅠ was positive), IgG–aCL, IgM–aCL, IgG–aβ_2_GPⅠ, and IgM–aβ2GPI in 1082 aPL-positive subjects.

The cross-positivity for aCL and aβ_2_GPI according to isotype in this study is shown in **Figure 1**. Of the aCL-positive subjects, only 3.4% (27/800) of them presented with isolated IgA–aCL. Similarly, of the aβ_2_GPI-positive subjects, isolated IgA–aβ_2_GPI only accounted for 3.7% (33/887) of the total. Further analysis of the isotype distribution between aCL and aβ_2_GPI showed that the proportion of IgA–aCL (either IgA–aCL or IgA–aβ_2_GPI was positive) of the positive patients (any isotype of aPL was positive) was merely 1.94% (21/1082).

### Prevalence of aPL in Patients With APS and Healthy Controls

Compared with healthy controls, the total positivity of aPL isotype-specific antibodies was significantly higher in patients with APS. For isolated positivity, the positive rate of IgA–aCL and IgA–aβ_2_GPI were observed no differences between APS and healthy controls (**Table 4**). A higher frequency of positivity in patients with SAPS compared to PAPS was observed for aCL (χ^2^ = 16.481, *P* < 0.001) and aβ_2_GPI (χ^2^ = 7.868, *P* = 0.005). The frequency of IgA–aCL was also higher in patients with SAPS than in PAPS patients (χ^2^ = 6.044, *P* = 0.02). The presence of IgA–aβ_2_GPI was detected in 10.46% of PAPS patients, and it was detected in 20.34% of SAPS patients; however, there was no statistical difference in the positive rate of IgA–aβ_2_GPI between PAPS patients and SAPS patients (χ^2^ = 3.627, *P* = 0.07). Merely three (1.96%) patients presented with isolated IgA–aCL in the APS group, and only one (1.7%) patient presented with isolated IgA–aCL in the SAPS group. For IgA–aβ_2_GPI, only one (0.65%) patient in the PAPS group presented with isolated positivity, while no isolated positivity was observed in the SAPS group.

**Table 4:** Profile of antiphospholipid antibodies in patients with APS or healthy controls.

	Total positivity							
	APS			Healthy controls (n=120)	χ_1_^2^	*P_1_*	χ_2_^2^	*P_2_*
	Total (n=212)	PAPS (n = 153)	SAPS (n = 59)					
aCL	118 (55.66%)	72 (47.06%)	46 (77.97%)	3 (2.5%)	93.49	<0.001	16.481	<0.001
IgA–aCL	33 (15.57%)	18 (11.76%)	15 (25.42%)	0	20.741	<0.001	6.044	0.020
IgG–aCL	106 (50%)	64 (41.83%)	42 (71.19%)	1 (0.83%)	84.807	<0.001	14.678	<0.001
IgM–aCL	19 (8.96%)	10 (6.54%)	9 (15.25%)	2 (1.67%)	6.883	0.009	3.967	0.060
aβ2GPI	122 (57.55%)	79 (51.64%)	43 (72.88%)	5 (4.17%)	92.441	<0.001	7.868	0.005
IgA–aβ_2_GPⅠ	28 (13.21%)	16 (10.46%)	12 (20.34%)	0	17.309	<0.001	3.627	0.070
IgG–aβ_2_GPⅠ	90 (42.45%)	56 (36.60%)	34 (57.63%)	0	69.889	<0.001	7.705	0.008
IgM–aβ2GPⅠ	57 (26.89%)	33 (21.57%)	24 (40.68%)	5 (4.17%)	26.045	<0.001	7.910	0.006
**Isolated positivity**								
aCL								
IgA–aCL	4 (1.89%)	3 (1.96%)	1 (1.70%)	0	2.292	0.130	0.016	0.899
IgG–aCL	72 (33.96%)	45 (29.41%)	27 (45.76%)	1 (0.83%)	49.028	<0.001	5.076	0.035
IgM–aCL	7 (3.3%)	5 (3.27%)	2 (3.39%)	2 (1.67%)	0.777	0.378	0.002	0.965
aβ2GPI								
IgA–aβ_2_GPⅠ	1 (0.47%)	1 (0.65%)	0	0	0.658	0.451	0.387	0.534
IgG–aβ_2_GPⅠ	45 (21.23%)	34 (22.22%)	11 (18.64%)	0	29.466	<0.001	0.326	0.583
IgM–aβ2GPⅠ	28 (13.21%)	20 (13.07%)	8 (13.56%)	5 (4.17%)	6.997	0.008	0.009	0.925

P1, APS vs. HC; P2, PAPS vs. SAPS.

**Figure 2:**
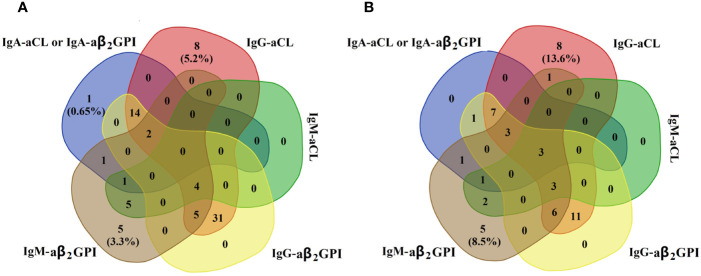
Distribution of IgA–aPL (either IgA–aCL or IgA–aβ_2_GPⅠ was positive), IgG–aCL, IgM–aCL, IgG–aβ_2_GPⅠ, and IgM–aβ2GPⅠ in the **(A)** PAPS group (n = 153) and **(B)** SAPS (n = 59).

We also observed cross-positivity in the prevalence of the aPL isotype in the two groups (**Figure 2**). We found that most PAPS or SAPS patients with IgA–aPL positivity were accompanied with IgG- or IgM–aPL. In contrast, isolated IgA–aPL was rare in patients with PAPS or SAPS.

### Diagnostic Performance Analysis of IgA–aCL and IgA–aβ_2_GPⅠ Antibodies in APS Patients

**Figure 3:**
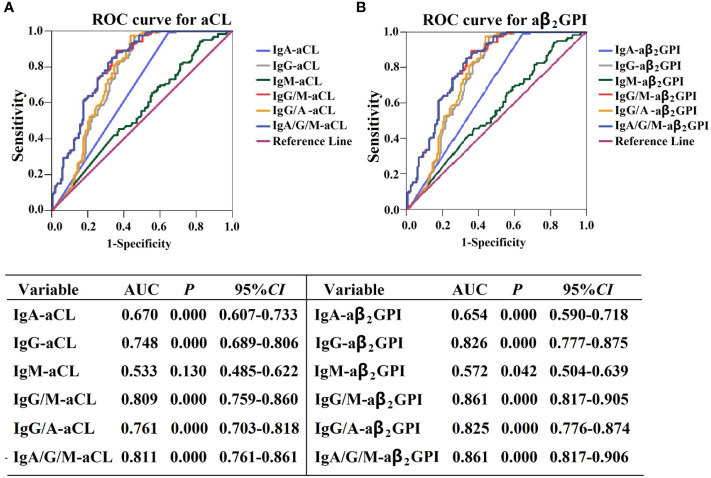
Receiver operating characteristic curve analysis of the aPL isotype in APS patients. **(A)** ROC curve for aCL and **(B)** ROC curve for aβ2GPI.

We performed ROC analysis to determine the diagnostic performance of aPL isotype-specific antibodies for APS patients. The AUC of IgA–aCL was 0.670 (95% CI: 0.607–0.733, *P* < 0.01), which was lower than that of IgG–aCL (AUC = 0.748, 95% CI: 0.689–0.806, *P* < 0.01). When IgA–aCL and IgG/M-aCL were merged for combination analysis, the AUC of IgG/M/A-aCL was 0.811 (95% CI: 0.761–0.861, *P* < 0.01), which was similar to that of IgG/M-aCL (AUC = 0.809, 95% CI: 0.759–0.860, *P* < 0.01) (**Figure 3A**). The AUC of IgA–aβ_2_GPI was 0.654 (95% CI: 0.590–0.718, *P* < 0.01), which was lower than that of IgG–aβ_2_GPI (AUC = 0.826, 95% CI: 0.777–0.875, *P* < 0.01). When IgA–aβ_2_GPI and IgG/M–aβ_2_GPI were merged for combination analysis, the AUC of IgG/M/A–aβ_2_GPI was 0.861 (95% CI: 0.817–0.905, *P* < 0.01), which was similar to that of IgG/M–aβ_2_GPI (AUC = 0.861, 95% CI: 0.817–0.906, *P* < 0.01) (**Figure 3B**). Overall, IgA–aPL antibodies alone or in combination with IgG–aPL or IgG/M–aPL antibodies did not increase the diagnostic efficiency in patients with APS.

### Associations Between APS-Related Clinical Manifestations of 212 Patients and the Presence of Each aPL Isotype

Among the various clinical manifestations presented by 212 APS, the presence of IgA–aPL wasn’t associated with any clinical manifestation. IgG–aPL was associated with cardiopulmonary involved [OR 30.512 (95% CI 1.969–472.780), P=0.015], acute coronary syndrome [OR 0.025 (0.001–0.906), P=0.025], pulmonary embolism [OR 0.058 (0.004–0.906), P=0.042] and thrombocytopenia [OR 1.992 (1.024–3.872), P=0.042]. Meanwhile, the results of IgM–aPL were different; the presence of IgM–aPL was associated with venous thrombosis [OR 3.078 (1.2–7.896), P=0.019] and thrombocytopenia [OR 4.977 (1.512–16.387), P=4.977]. The results are summarized in detail in **Table 5**.

**Table 5:** Associations between APS-related clinical manifestations of 212 patients with aPL levels.

	IgA				IgG				IgM			
	IgA–aPL positive (n = 34)	IgA–aPL negative (n = 178)	*P*	OR (95% CI)	IgG–aPL positive (n = 107)	IgG–aPL negative (n = 105)	*P*	OR (95% CI)	IgM–aPL positive (n = 57)	IgM–aPL negative (n = 155)	*P*	OR (95% CI)
PAPS	19 (55.88%)	134 (75.28%)	0.067	0.44 (0.183–1.061)	64 (59.81%)	89 (84.76%)	0.001	0.259 (0.118–0.565)	33 (57.89%)	120 (77.42%)	0.056	0.473 (0.219–1.020)
SAPS	15 (44.12%)	44 (24.72%)			43 (40.19%)	16 (15.24%)			24 (42.12%)	35 (22.58%)		
Arterial thrombosis	17 (50.00%)	60 (33.71%)	0.464	1.67 (0.423–6.599)	45 (42.06%)	32 (30.48%)	0.540	1.388 (0.486–3.964)	22 (38.60%)	55 (35.48%)	0.405	1.594 (0.532–4.778)
Venous thrombosis	17 (50.00%)	87 (48.88%)	0.229	1.948 (0.657–5.773)	58 (54.21%)	46 (43.81%)	0.175	1.861 (0.758–4.568)	35 (61.40%)	69 (44.52%)	0.019	3.078 (1.200–7.896)
Pregnancy morbidity	15 (44.12%)	70 (39.32%)	0.056	2.779 (0.973–7.942)	36 (33.64%)	49 (46.67%)	0.896	1.061 (0.437–2.580)	20 (35.09%)	65 (41.94%)	0.401	1.485 (0.590–3.733)
Stroke	11 (32.35%)	27 (15.17%)	0.600	1.468 (0.350–6.163)	25 (23.36%)	13 (12.38%)	0.692	1.3 (0.356–4.753)	9 (15.79%)	29 (18.71%)	0.597	1.456 (0.361–5.881)
Cardiopulmonary involved	10 (29.41%)	42 (23.60%)	0.478	0.674 (0.227–2.005)	32 (29.91%)	20 (19.00%)	0.015	30.512 (1.969–472.780)	13 (22.81%)	39 (25.16%)	0.401	0.351 (0.031–4.034)
Acute coronary syndrome	1 (2.94%)	6 (3.37%)	0.887	0.826 (0.059–11.526)	2 (1.87%)	5 (4.76%)	0.025	0.025 (0.001–0.641)	1 (1.75%)	6 (3.87%)	0.852	1.279 (0.097–16.909)
Pulmonary embolism	8 (23.53%)	34 (19.1%)	0.561	1.562 (0.347–7.042)	25 (23.36%)	17 (16.19%)	0.042	0.058 (0.004–0.906)	12 (21.05%)	30 (19.35%)	0.909	1.148 (0.107–12.315)
Valvular lesions	3 (8.82%)	6 (3.37%)	0.365	2.374 (0.366–15.382)	6 (5.61%)	3 (2.86%)	0.113	0.186 (0.023–1.487)	1 (1.75%)	8 (5.16%)	0.394	0.32 (0.023–4.388)
Renal involved	6 (17.65%)	20 (11.24%)	0.900	1.075 (0.348–3.318)	18 (16.82%)	8 (7.62%)	0.319	1.673 (0.608–4.607)	9 (15.79%)	17 (10.97%)	0.797	1.15 (0.395–3.345)
Autoimmune hemolytic anemia	4 (11.77%)	15 (8.43%)	0.498	0.62 (0.156–2.472)	11 (10.28%)	8 (7.62%)	0.160	0.431 (0.133–1.395)	12 (21.05%)	7 (4.52%)	0.008	4.977 (1.512–16.387)
Thrombocytopenia	22 (64.71%)	78 (43.82%)	0.146	1.922 (0.797–4.633)	63 (58.88%)	37 (35.24%)	0.042	1.992 (1.024–3.872)	30 (52.63%)	70 (45.16%)	0.484	0.769 (0.367–1.607)

## Discussion

APS is an autoimmune disease that affects nearly 5 in 100,000 people each year (24). aPL is a critical laboratory biomarker for the diagnosis of APS. IgA–aPL, also known as a “non-criteria” aPL, has been shown to have a controversial diagnosis performance for APS in previous studies (19, 20, 25) and a systematic review (26). To the best of our knowledge, this is the first study to analyze the aPL profile based on 7,293 consecutive subjects from a large cohort in the general Chinese population. We took advantage of this unselected large cohort and were able to analyze the relationship between the IgA isotype of aPL and the IgG/M isotype of aPL in the general population as well as in APS patients. We demonstrated that extra IgA–aPL testing did not provide any additional value for the diagnosis of APS.

In the current study, we found that the prevalences of IgA–aCL and IgA–aβ_2_GPI were 2.48 and 2.13%, respectively, in the general population. According to previous research, the prevalence of IgA–aCL ranges from 1.6 to 10% (27–30), and the prevalence of IgA–aβ_2_GPI ranges from 3 to 20.8% (29–32). These findings are consistent with our data. We also evaluated cross-positivity for the aPL profile in the general population when IgA–aCL was combined with IgA–aβ_2_GPI as a group; only 21 (0.29%) subjects presented isolated IgA–aPL, indicating that IgA–aPL is commonly accompanied with IgG–aPL and/or IgM–aPL and that it is rarely isolated in the general population.

We identified the patients diagnosed with APS in our cohort. Of patients with PAPS, the results showed that the proportion of those with IgA–aCL was 11.76%, and the proportion of those with IgA–aβ_2_GPI was 10.46%; while it was 25.42 and 20.34%, respectively, in patients with SAPS. The prevalence of IgA–aPL was consistent with that reported by the Antiphospholipid Syndrome Alliance for Clinical Trials and International Networking (33), a Swedish report (17), and the Hopkins Lupus Cohort (12).

Although the presence of IgA–aPL is not rare in PAPS or SAPS, cases with isolated IgA–aPL should receive more attention due to its clinical relevance. It stands out that only one case (0.65%) of our patients with PAPS had isolated IgA–aPL, but there were no cases of isolated IgA–aPL among the SAPS patients. These results indicate that IgA–aPL is usually accompanied by IgG/M–aPL and that isolated IgA–aPL is rare in patients with APS. Different from previous researches results, the prevalence of isolated IgA–aPL in Frodlund M (17) and Ruiz-Garcia R (25) were higher than that in this study, possible seasons for this difference should due to the patients population and detection assay used.

ROC curve analysis revealed that IgA–aCL and IgA–aβ_2_GPI have a similar diagnostic accuracy for APS, with AUCs of 0.670 (95%CI: 0.607–0.733, *P* < 0.001) and 0.654 (95%CI: 0.590–0.718, *P* < 0.001), respectively. It is worth noting that the triple detection of IgA/G/M-aCL or IgA/G/M–aβ_2_GPI does not increase the diagnostic performance of APS compared with the aPL detection criteria such as IgG isotype alone or in combination with the IgG or IgM isotype. Consistently, several previous studies have failed to show that adding the detection of IgA–aCL or IgA–aβ_2_GPI increases the diagnostic performance (20, 34–38). The uselessness of the aPL isotype triple detection is probably due to the relatively low prevalence of IgA–aPL and the fact that most cases of IgA–aPL were accompanied with another isotype of aPL. The diagnostic accuracy of routine detection and the correlation between IgA–aPL and APS-associated clinical manifestation could also be considered as other possible reasons.

According to the recent study, whether it is clinical manifestation criteria, mainly including arterial or venous thrombosis and pregnancy morbidity, or some other “non-criteria” clinical manifestations such as stroke and thrombocytopenia, there is no correlation between IgA–aPL positivity and clinical manifestations. In line with the previously published large cohort studies of APS patients, the results showed no association between IgA–aβ_2_GPI and APS manifestations in APS patients (18, 19). Of note, whether in a laboratory or clinical setting, the detection of IgA–aPL does not provide more value for the diagnosis of APS as it can be replaced by IgG–aPL detection. However, a recent study has revealed the opposite conclusion that IgA–aPL is associated with thrombosis and obstetric complications (10). Some other previous studies also have mentioned the robust association between IgA–aPL and thrombosis (13, 39–42) and that even IgA–aβ_2_GPI can be used as an independent risk factor for the development of APS-related events (13) and should be included as a consensus criterion for the diagnosis of APS (43). These controversial conclusions can mainly be attributed to differences in the included criteria of the study population, ethnic distribution, or statistical methods of the study. It is important to note that the proportion of isolated IgA–aPL was extremely low in our study or in controversial study (10). These findings reveal that the clinical relevance of IgA–aPL to APS is limited and uncertain. Therefore, whether a “real world” relationship between IgA–aPL and APS-related manifestations exists or whether IgA–aPL can be used as a diagnostic criterion in patients with APS requires further study.

As indicated by our results, whether it is for IgM–aCL or IgM–aβ2GPI, the AUCs were lower than those of IgG–aPL and IgA–aPL; however, the diagnostic efficacy for APS was improved when IgM–aPL was used in combination with the IgG isotype. Moreover, unlike IgA–aPL, IgM–aPL is directly related to venous thrombosis and autoimmune hemolytic anemia. A previous study has suggested an increased risk of retinal thrombosis in isolated IgM isotype-positive APS patients (44). Therefore, IgM–aPL can identify different types of APS manifestations in addition to being a diagnostic standard related to clinical manifestation criteria; it also can be used as a supplement for IgG–aPL.

There are several limitations to our study. Some of them are inherent to the overall population. Although the investigated cohort of this study was subjects with clinical suspicion of APS, both naive and follow-up patients were included in this research. Some characteristics of the patients who were followed up for several months might have changed compared to those at the first detection of aPL. Thus, our results did not reflect the true state of IgA–aPL of patients in the initial situation. Additionally, the lack of consecutive aPL profile detection on the same patients makes it impossible to estimate the clinical value of IgA–aPL for the diagnosis of APS over time. Besides, the sensitivity and diagnostic efficiency may be variable using different detection system or kits (45), which are inherent to the methodology or the selection of the antigen target (20, 46–48). These limitations will be the focus of a future study.

In conclusion, IgA–aPL is present in a small proportion of the general population. Besides, most IgA–aPL was accompanied with the IgG and/or IgM isotype of aPL, and isolated IgA–aPL rarely appeared in the general population and APS patients. Thus, extra IgA–aPL testing does not improve the diagnostic performance of aPL for APS. Moreover, the presence of IgA–aPL is not associated with clinical manifestation of APS. From a clinical point of view, these data do not support the utility of IgA–aPL as an additional biomarker for the diagnosis of APS, and IgA–aPL is not recommended as a diagnostic criterion for APS. However, additional highly sensitive aPL testing assays are needed to confirm the characteristics of different ethnic groups with IgA–aPL as well as in a large cohort of APS patients.

## Data Availability Statement

All datasets presented in this study are included in the article/supplementary material.

## Ethics Statement

This study was approved by the Medical Ethics Committee of PUMCH, and all methods were carried out in accordance with the principles stated in the Declaration of Helsinki. The patients/participants provided their written informed consent to participate in this study.

## Author Contributions

All authors were involved in the design of this study. CH, XL, and JZ were involved in the collection of blood samples, experimental procedures, statistical analysis, and writing of the manuscript. QW, ML, XT, XZ, and JZ were involved in the recruitment of patients and evaluation of clinical data. All authors contributed to the article and approved the submitted version.

## Funding

This study was supported by the National Key Research and Development Program of China (2019YFC0840603, 2017YFC0907601, and 2017YFC0907602), the National Natural Science Foundation of China (81771780), and the CAMS Initiative for Innovative Medicine (2017-I2M-3-001 and 2019-I2M-2-008).

## Conflict of Interest

The authors declare that the research was conducted in the absence of any commercial or financial relationships that could be construed as a potential conflict of interest.
